# Promoter Variation in the DC-SIGN–Encoding Gene *CD209* Is Associated with Tuberculosis

**DOI:** 10.1371/journal.pmed.0030020

**Published:** 2006-01-03

**Authors:** Luis B Barreiro, Olivier Neyrolles, Chantal L Babb, Ludovic Tailleux, Hélène Quach, Ken McElreavey, Paul D. van Helden, Eileen G Hoal, Brigitte Gicquel, Lluis Quintana-Murci

**Affiliations:** 1CNRS FRE2849, Unit of Molecular Prevention and Therapy of Human Diseases, Institut Pasteur, Paris, France; **2**Unité de Génétique Mycobactérienne, Institut Pasteur, Paris, France; **3**Faculty of Health Sciences, Stellenbosch University, Tygerberg, South Africa; **4**Reproduction, Fertilité et Populations, Institut Pasteur, Paris, France

## Abstract

**Background:**

Tuberculosis, which is caused by *Mycobacterium tuberculosis,* remains one of the leading causes of mortality worldwide. The C-type lectin DC-SIGN is known to be the major *M. tuberculosis* receptor on human dendritic cells. We reasoned that if DC-SIGN interacts with *M. tuberculosis,* as well as with other pathogens, variation in this gene might have a broad range of influence in the pathogenesis of a number of infectious diseases, including tuberculosis.

**Methods and Findings:**

We tested whether polymorphisms in *CD209,* the gene encoding DC-SIGN, are associated with susceptibility to tuberculosis through sequencing and genotyping analyses in a South African cohort. After exclusion of significant population stratification in our cohort, we observed an association between two *CD209* promoter variants (−871G and −336A) and decreased risk of developing tuberculosis. By looking at the geographical distribution of these variants, we observed that their allelic combination is mainly confined to Eurasian populations.

**Conclusions:**

Our observations suggest that the two −871G and −336A variants confer protection against tuberculosis. In addition, the geographic distribution of these two alleles, together with their phylogenetic status, suggest that they may have increased in frequency in non-African populations as a result of host genetic adaptation to a longer history of exposure to tuberculosis. Further characterization of the biological consequences of DC-SIGN variation in tuberculosis will be crucial to better appreciate the role of this lectin in interactions between the host immune system and the tubercle bacillus as well as other pathogens.

## Abstract 2

Two variants in the gene that encodes DC-SIGN, the major receptor for *Mycobacterium tuberculosis* on dendritic cells, are associated with susceptibility to TB

## Introduction

One-third of the world's population is estimated to be infected with *Mycobacterium tuberculosis,* the etiological agent of tuberculosis (TB). This disease tops the World Health Organization list of deaths due to a single infectious agent, with the death toll between 2 and 3 million people per year [1]. A perplexing, and yet unsolved, feature of TB is that less than 10% of infected individuals develop the disease. Substantial epidemiological evidence supports that host-related factors, such as sex, age, HIV infection, malnutrition, and BCG (bacille Calmette-Guérin) vaccination, influence the balance between the tubercle bacilli and host immune defences [2,3]. In addition, there is increasing evidence that host genetic factors determine differences in host susceptibility to mycobacterial infection and might contribute therefore to the pattern of clinical disease [4–7]. From a host perspective, the innate immunity system acts as the first line of host defense against microbial pathogens [8]. Initial recognition of pathogens by the innate immunity system is mediated by phagocytic cells, such as dendritic cells (DCs) or macrophages, through germline-encoded receptors, known as pattern recognition receptors [9]. DCs bear a range of pattern recognition receptors, such as C-type lectins and Toll-like receptors, involved both in recognition of conserved products of microbial metabolism and in the induction of adaptive immunity [8,10–12]. In particular, C-type lectins detect pathogens by their characteristic carbohydrate structures and internalise them for further antigen processing and presentation [13]. We have recently shown that a prototypic C-type lectin, DC-SIGN (dendritic cell–specific ICAM-3 grabbing nonintegrin), is the major *Mycobacterium tuberculosis* receptor on human DCs [14]. DC-SIGN is specifically, though not exclusively, expressed on DCs and functions both as a cell adhesion and as a pathogen recognition receptor [15]. As an adhesion receptor, it plays an important role in many DC functions, such as DC-T cell interaction and DC migration [16,17]. Besides its cellular recognition role, DC-SIGN serves as pathogen uptake receptor and mediates interactions with a plethora of pathogens other than *M. tuberculosis* [18]. Indeed, it has been shown that DC-SIGN allows DCs to capture other bacteria such as *Helicobacter pylori* and certain *Klebsiella pneumonia* strains, but also viruses such as HIV-1, Ebola, cytomegalovirus, hepatitis-C, dengue, and SARS-coV, and parasites like *Leishmania pifanoi* and *Schistosoma mansoni* [19–27]. In addition, recent data suggest that DC-SIGN may mediate intracellular signalling events leading to cytokine secretion and, on this basis, it has been proposed that the lectin could be used by pathogens, including *M. tuberculosis,* as a part of an immune evasion strategy to their own advantage [28,29].

In light of the ability of DC-SIGN to interact with *M. tuberculosis* and other pathogens, it is plausible that variation in its gene may influence the pathogenesis of a number of infectious diseases, including TB. We have therefore explored the relationship between *CD209* polymorphisms and susceptibility to TB by determining *CD209* sequence variation in a cohort of South African Coloured origin.

## Methods

### Patients and Methods

The study was conducted in a cohort of 711 individuals, including 351 TB patients and 360 healthy controls, living in the Cape Town area. Certain suburbs of metropolitan Cape Town have some of the highest reported incidence rates of TB in the world, despite extensive BCG vaccination. Indeed, our study population comes from two suburbs that have been extensively studied because of their uniform ethnicity (known as South African Coloured) and socio-economic status as well as high incidence of TB and low prevalence of HIV [30]. In addition, our study group represents a present-day homogenous population [31] that previously received genetic input from Khoisan, Malaysian, Bantu, and European descent populations [32]. Thus, it represents a community originating from populations with different susceptibilities to TB and offers a unique opportunity to dissect the contributing genetic variants and their probable geographic/ethnic origins. TB patients were bacteriologically-confirmed (smear-positive and/or culture-positive) to present pulmonary tuberculosis. Their mean age (± standard deviation) was 36.7 ± 10.9 y, and 51.8% were male. Controls were unrelated healthy individuals from the same community, with the same socio-economic status, access to health facilities, and chance of diagnosis, and with neither signs nor previous history of TB (mean age 34.6 ± 12.5 y, 22% male). The annual risk of infection in this suburb was estimated at 2.5% in 1987 and at 2.8%–3.5% in 1999, and it is therefore highly likely that, in such an environment, the vast majority of controls have been exposed to *M. tuberculosis* [33,34]. All subjects were HIV-negative and older than 18 y. Informed consent was obtained from all participants, and the study was approved by the ethics committee of the Faculty of Health Sciences, Stellenbosch University (South Africa).

### Laboratory Procedures and Statistical Analysis

To identify informative *CD209* single nucleotide polymorphisms (SNPs) and to avoid ascertainment bias in the choice of markers to be tested, we first sequenced the whole *CD209* genomic region (seven coding exons, flanking intronic regions, and 1,000 base pairs situated 5′ of the start codon) in 28 randomly chosen individuals (56 chromosomes). Using polymorphisms with a minimum allele frequency of 0.05, unphased genotypic data were converted into haplotypes using the accelerated EM (Expectation Maximization) algorithm implemented in Haploview v3.1 [35]. To evaluate the accuracy of the EM algorithm, haplotype reconstruction was performed in parallel using the Bayesian statistical method [36] implemented in Phase v.2.1.1. Equivalent results were obtained using both methods, with all haplotypes presenting high levels of statistical support. In order to define a minimal number of SNPs explaining most haplotypic diversity, we used the BEST v1.0 software [37]. Eight haplotype-tagging SNPs were then selected to genotype the entire panel of 711 individuals. Further, potential population stratification between cases and controls was tested by genotyping 25 unlinked SNP markers in the entire study cohort. DNA samples were genotyped by either fluorescence polarization (VICTOR-2TM technology; PerkinElmer, Wellesley, California, United States) or TaqMan (ABI Prism-7000 Sequence Detection System; Applied Biosystems, Foster City, California, United States) assays. Statistical testing for genotypic and haplotypic associations were performed using S TATA 8.2 and Haploview v3.1, respectively. The haplotype frequencies were obtained by summing the fractional likelihood of each haplotype for each individual (i.e., if a particular individual has been determined to have a 40% likelihood of haplotype A and 60% likelihood of haplotype B, 0.4 and 0.6 would be added to the counts for A and B, respectively) [35].

## Results and Discussion

Two variants located in the *CD209* promoter region (−871 A/G and −336 A/G) exhibited a frequency distribution significantly distorted between TB patients and controls, as indicated by a Chi-square test (Table 1). For the −871 variant, genotypes GG and GA were less frequently observed in cases (16.8%) compared to the control group (27.2%) (*p* = 8.2 × 10^−4^). For the −336 variant, genotypes GG and GA were more frequent in cases (70.6%) than in controls (61.9%) (*p* = 0.01). These observations suggest that the alleles −871A (odds ratio [OR]: 1.85; 95% CI: 1.29–2.66) and −336G (OR: 1.48; 95% CI: 1.08–2.02) increase the risk of developing TB in our South African cohort. At the haplotype level (Table 2), a Chi-square test first revealed that the global distribution of haplotype frequencies was significantly different between cases and controls (*p* = 1.2 × 10^−3^). One haplotype (H3) turned out to be the main haplotype responsible for such a distorted frequency distribution (Table 2). This haplotype, which contains both −871G and −336A, was found to be strongly associated with the control group (*p* = 1.6 × 10^−3^; OR: 1.7; 95% CI: 1.22–2.38). The associations with this haplotype, and with −871, remained highly significant (*p* = 1.3 × 10^−2^ and 6.6 × 10^−3^ respectively), even after the conservative Bonferroni correction for multiple testing.

**Table 1:**
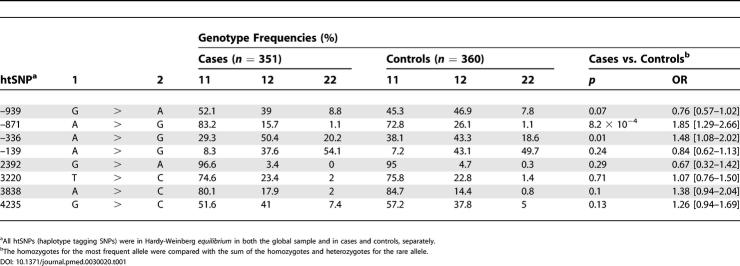
DC-SIGN Genotype Distributions in Patients with Tuberculosis and in Healthy Controls

**Table 2:**
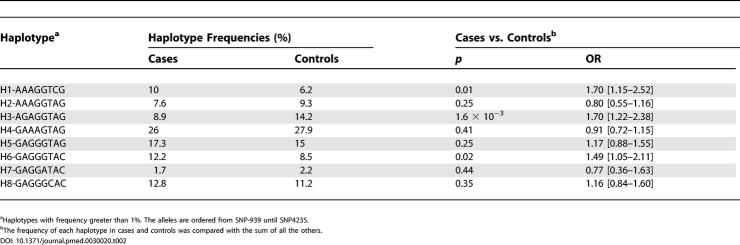
Haplotype Distributions in Patients with Tuberculosis and in Healthy Controls

Although our cohort is considered a present-day homogeneous community that has received genetic contribution from different populations multiple generations ago [31,32], population stratification between cases and controls can be a confounding factor leading to a spurious positive association. Indeed, the use of admixed populations in association-mapping studies can be very useful to identify disease-causing genetic variants that differ in frequency across parental populations. However, when the admixture event is too recent, allelic frequencies can differ coincidentally among cases and controls, reflecting a nonuniform genetic contribution from the parental populations to each subpopulation (i.e., cases and controls), rather than a genuine association between a given genetic variant and the phenotype under study. In this case, the study-cohort is said to present population stratification. To formally test and quantify the levels of background genetic differences [38], if any, between cases and controls, we genotyped the entire cohort for a panel of 25 independent SNPs markers which are (1) not in linkage disequilibrium with the candidate *CD209* locus and with any other known gene, (2) randomly distributed along the genome, and (3) polymorphic among the major ethnic groups (Table 3). The mean χ^2^ statistic among the 25 SNPs for the comparison of allele frequencies between cases and controls, which represents the levels of stratification (μ) between the two groups [39], was 1.25 (*p* = 0.26), implying that the two groups were not significantly stratified. As an additional correction for stratification, we divided the χ^2^ values obtained for our candidate gene *CD209* by the level of stratification detected (1.25) [39]. Even after such a conservative correction, the associations observed with −336 and −871 as well as with H3 remained significant (−336 *p* = 2.8 × 10^−2^; −871 *p* = 2.7 × 10^−3^; H3 *p* = 4.8 × 10^−3^). These observations support therefore the idea that the −871G and −336A variants are indeed genuinely associated with a protective role against TB.

**Table 3:**
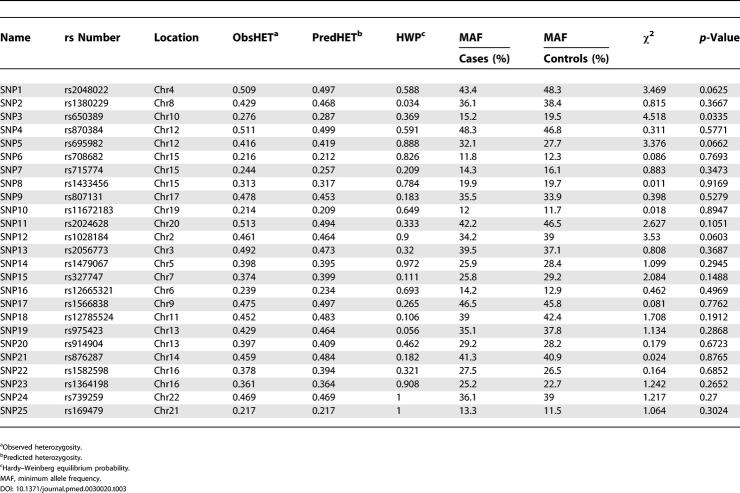
Frequency Distribution in the Study Cohort of the 25 SNPs Used to Test for Population Stratification

In order to gain insights into the frequency distribution of these two SNPs, we genotyped them in 254 human chromosomes from sub-Saharan Africa, Europe, and East Asia as well as in eight chimpanzee chromosomes. We observed that the −871G and −336A forms, which we propose as offering protection against TB, corresponded to the derived allele in humans; we also observed that these forms are present at higher frequencies in Eurasians as compared to Africans (Table 4). Indeed, the −871G is absent in African populations whereas it reaches high frequencies (20%–40%) in European and Asian populations. Given the absence of the haplotypic combination of −871G and −336A among sub-Saharan Africans, its presence among South African Coloureds suggests that it was introduced through the historically well-known admixture with Europeans and Asians [31]. This observation highlights the power of using admixed populations to better understand historical issues associated with the geographic/ethnic origin of disease-affecting alleles, provided that their prevalence varies in the ancestors of the admixed population (i.e., different frequency of H3 in Africans versus non-Africans; Table 4).

**Table 4:**
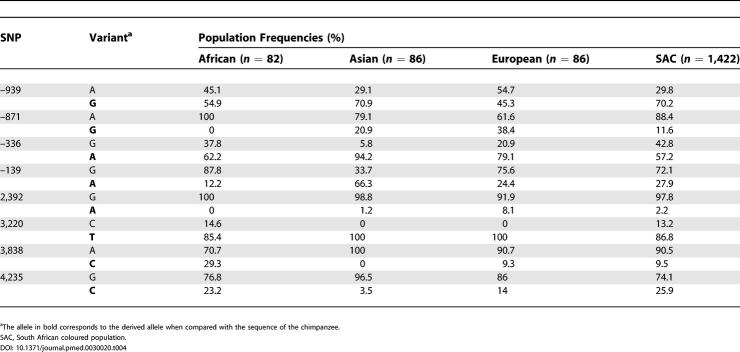
Frequency Distribution of the Eight *CD209* SNPs Genotyped in the Multi-Ethnic Panel of 127 Individuals (*n* = 254 Chromosomes) as Well as in the 711 Individuals of the South African Cohort (*n* = 1,422 Chromosomes)

In the context of TB, it has been suggested that present-day susceptibility to TB is determined by previous history of exposure [40]. There is fairly convincing evidence that TB has been endemic in Europe for several hundred years, whereas in Africa it has probably been rare before contact was initiated with Europeans [41–43]. It is expected therefore that *M. tuberculosis* has exerted stronger selective pressures on European than African populations [42]. Our results lend support to this hypothesis and suggest that the protective alleles −871G and −336A increased in frequency in non-African populations as a result of genetic adaptation to a longer period of TB exposure. The potential impact of tuberculosis on the frequency of resistant alleles in European populations has been recently addressed using epidemiological data and statistical modeling [44]. The authors have sought to evaluate the expected changes in resistant allele frequencies, during the 300-y period corresponding to the peak epidemics of TB in Europe. They concluded that if a given resistant allele was at a low frequency in the beginning of an epidemic, selection by *M. tuberculosis* alone would increase the frequency of this allele, but not enough to bring it to epidemiologically significant levels. In this context, since DC-SIGN is known to interact with a vast range of pathogens, it is indeed likely that the increased frequencies observed today for both −871G and −336A in non-African populations (specially for −871G which is absent in sub-Saharan Africans) may have been driven, not only by the selective pressures imposed by *M. tuberculosis,* but also by other infectious agents. Indeed, two independent studies have recently reported a genetic association between the −336A variant and protection against parenteral HIV infection [45] and severity of dengue pathogenesis [46]. Although HIV infection, for example, is too recent to have left any signature of selection on *CD209,* these observations emphasize the possible action of other pathogens in shaping the patterns of variability of this gene.

From a functional point of view, the −336A allele has been shown to affect an Sp1-like binding site and to modulate transcriptional activity in vitro by increasing the levels of expression [46]. In the context of TB, increased DC-SIGN expression levels by DCs may result in better capture and processing of mycobacterial antigens, leading to a stronger and wider T-cell response. In addition, we have recently shown that DC-SIGN expression is markedly induced in alveolar macrophages in active TB patients and that *M. tuberculosis* is preferentially phagocytosed by DC-SIGN–expressing macrophages in these individuals [47]. Thus, the higher prevalence observed among healthy individuals of the −336A variant, which is associated with increased DC-SIGN expression, may underlie an increased efficiency of host phagocytes, such as DCs and macrophages, to control the infection. In addition to the −336A variant, our genetic data showed a strong association of the −871G allele with healthy controls, suggesting also a functional consequence of this variant that, either alone or in combination with −336A, remains to be defined.

In conclusion, the significant association found for the *CD209* promoter variants together with their phylogenetic status and frequency distribution strongly suggests that the −871G and −336A alleles may reduce the risk of developing TB. More generally, our results, together with those reporting association of *CD209* promoter variants with both HIV susceptibility and dengue pathogenesis [45,46] suggest that variation in this lectin may be of crucial importance in the outcome of a number of infections due to DC-SIGN–interacting pathogens. Detailed in vitro and in vivo studies assessing the functional consequences of *CD209* variants on the quality of the host immune response against pathogens, including *M. tuberculosis,* are now required to eventually develop knowledge-based and effective pathway-targeted treatments.

**Patient Summary**

**Background**

Tuberculosis (TB) is an infectious disease caused by the bacterium *Mycobacterium tuberculosis*. The disease kills between 2 million and 3 million people each year, and almost one-third of the world's population, or 2 billion people, are thought to be infected with the bacterium. However, only about 10% of infected individuals become sick. In the other cases, the body's immune system is capable of controlling the growth of the bacteria. Whether a person's immune system is strong enough to keep the bacterium in check depends on a variety of factors, such as age, how well nourished the person is, other infections, and genetic make-up.

**Why Was This Study Done?**

Understanding the genetic factors that influence whether an infected person is more or less susceptible to getting sick with TB should help doctors to fight the disease. A number of research groups are studying these genetic factors, and some genes have previously been identified that seem to increase a person's risk of TB. Some of the authors of this study work on a molecule that is part of the immune system. The molecule is called DC-SIGN, and it plays a role in infection of cells in the lung by the Mycobacterium. Together with colleagues who are genetics experts, they wanted to see whether variation in the gene (called *CD209*) that codes for DC-SIGN influences the risk of someone infected with *M. tuberculosis* getting sick.

**What Did the Researchers Do and Find?**

They worked with a group of South African participants from an area close to Cape Town where TB is very common. Half of the participants had TB; the other half did not (even though, because TB is so common in that area, it was likely that most if not all of them had been infected with *M. tuberculosis* at some point). The researchers compared make-up of the gene for DC-SIGN in all participants and found that some variants of the gene were more common in the group of healthy individuals. In other words, having this particular type of DC-SIGN-encoding gene seemed to protect individuals from getting TB.

**What Does This Mean?**

DC-SIGN is a central part of the immune system, and others scientists have reported links between variants in the gene for DC-SIGN and the risk of picking up other infectious diseases, including dengue fever and HIV/AIDS. The present study lends more support to the notion that DC-SIGN is a key player in the control of infectious diseases. Understanding more about DC-SIGN could help to develop better treatments for these infections.

**Where Can I Find More Information Online?**

The following Web sites provide information about tuberculosis.

World Health Organization pages on TB:

http://www.who.int/mediacentre/factsheets/fs104/en/index.html

OMNI pages on TB:

http://omni.ac.uk/browse/mesh/D014376.html

Medline PLUS pages on TB:

http://medlineplus.nlm.nih.gov/medlineplus/tuberculosis.html

European TB Vaccine Cluster:

http://www.tb-vac.org

## Abbreviations

DC: dendritic cell
OR: odds ratio
SNP: single nucleotide polymorphism
TB: tuberculosis
